# A modular, deep learning-based holistic intent sensing system tested with Parkinson’s disease patients and controls

**DOI:** 10.3389/fneur.2023.1260445

**Published:** 2023-11-01

**Authors:** Joseph Russell, Jemma Inches, Camille B. Carroll, Jeroen H. M. Bergmann

**Affiliations:** ^1^Natural Interaction Lab, Department of Engineering Science, Institute of Biomedical Engineering, University of Oxford, Oxford, United Kingdom; ^2^University Hospitals Plymouth NHS Trust, Plymouth, Devon, United Kingdom; ^3^Newcastle University Translational and Clinical Research Institute, Campus for Ageing and Vitality, Newcastle-upon-Tyne, United Kingdom; ^4^Faculty of Health, University of Plymouth, Plymouth, United Kingdom

**Keywords:** Parkinson’s disease, wearable sensors, intent sensing, deep learning, assistive medical devices

## Abstract

People living with mobility-limiting conditions such as Parkinson’s disease can struggle to physically complete intended tasks. Intent-sensing technology can measure and even predict these intended tasks, such that assistive technology could help a user to safely complete them. In prior research, algorithmic systems have been proposed, developed and tested for measuring user intent through a Probabilistic Sensor Network, allowing multiple sensors to be dynamically combined in a modular fashion. A time-segmented deep-learning system has also been presented to predict intent continuously. This study combines these principles, and so proposes, develops and tests a novel algorithm for multi-modal intent sensing, combining measurements from IMU sensors with those from a microphone and interpreting the outputs using time-segmented deep learning. It is tested on a new data set consisting of a mix of non-disabled control volunteers and participants with Parkinson’s disease, and used to classify three activities of daily living as quickly and accurately as possible. Results showed intent could be determined with an accuracy of 97.4% within 0.5 s of inception of the idea to act, which subsequently improved monotonically to a maximum of 99.9918% over the course of the activity. This evidence supports the conclusion that intent sensing is viable as a potential input for assistive medical devices.

## 1. Introduction

Parkinson’s disease is a neurodegenerative disease resulting in, among other symptoms, a gradual impairment of the patient’s mobility and quality of life (1). However, it often does not initially severely impair patients’ cognitive functions (2), meaning people living with it can report that they find themselves no longer physically able to complete tasks they might still mentally intend to do (3). Assistive technologies, such as tremor-suppressing wearables (4) or motion supporting exoskeletons (5), can help people complete tasks that they previously were unable to.

The loss of physical control caused by the disease, while still maintaining cognitive functions and therefore action intent, makes Parkinson’s disease patients an ideal target group for intent sensing – the prediction and measurement of what it is that a user wants to do (6). This paper proposes that intent sensing could be a useful input for control of assistive devices to help those living with Parkinson’s disease maintain their quality of life. It has been shown in previous studies to be an effective tool for the control of upper limb orthoses which assist motion (7), and provide intelligent attitude-adjustment for smart wheelchairs (8). These technologies can be applied for the support of patients with Parkinson’s disease, highlighting the potential developing intent technology for helping those with this condition.

It has been established in prior work (9) that intent sensing must be performed continuously over time, predicting an upcoming activity, detecting the activity’s onset, and monitoring the activity as it takes place and inferring its task goal. It was shown that the accuracy of intent prediction increases monotonically over time – predicting an activity before it starts is intuitively far more difficult than classifying it after it has been completed. An effective intent-sensing system should predict possible upcoming activities in advance, and then refine these predictions as the activity begins and progresses.

To minimise risk and maximise compliance (10), intent prediction should be performed non-invasively (unless the patient already has an implanted device). Information that can be used for prediction can be obtained from a range of sensors. Measurements from wearable and non-wearable sensors can be individually classified using deep learning (9), before being combined as a probabilistic sensor network to accurately determine user intent.

To ensure robustness and independence between sensors, multiple sensing modalities should be used (11). Many possible sensing modalities have been explored for intent, including electromyography (EMG), electroencephalography (EEG) and gaze-tracking (6). This study, however, will focus on motion data from Inertial Measurement Units (IMUs) and audio data from a microphone, as these modalities are representative of what might be found in typical consumer devices such as smart-watches and smart-phones (12), and are included in currently available wearable Parkinson’s disease-monitoring devices such as the Kinesia 360 (13).

Prior work has shown that there are many benefits to constructing an intent-sensing Probabilistic Sensor Network (14) using a modular method. They allow sensors to be freely added and removed from the network as they become available, without any retraining being required. This enables the possibility of a system where a user can move around a smart environment and take advantage of any wearable and non-wearable sensors they may encounter at any given time to always produce the most accurate prediction of intent.

Modular methods have also been shown to be far more robust to sensor unavailability, due to causes such as failure or, in the case of wearable sensors such as Surface EMG, sensor lift-off (11).

The benefit of modularity that this study will focus on, however, is the ability to add sensors to a network without increasing the complexity of the learned models, and therefore without requiring an exponentially increasing amount of data to properly train them.

To elaborate – if each sensor provides 18 features, and there are six sensors, as in this study, then combining all the features from all the sensors to train a single classifier requires learning of a model in 108 dimensions. Attempting to do this with only a small amount of training data will lead to overfitting, as separating data in that many dimensions is very easy for a classifier to do “by chance,” without learning any actual pattern that will reoccur for data that is not part of the training set.

This study, however, proposes to instead train one deep-learning classifier for each of the six sensors. With this approach, each classifier learned is only 18-dimensional, requiring much less training data to avoid overfitting. However, the same number of training data points are available as there were for the 108-dimensional classifier; it is simply the number of features that are reduced. As such, six much more effective classifiers are able to be learned, without discarding any of the features which may contain relevant information. The predictions from each of these classifiers can then be combined as part of a Probabilistic Sensor Network.

A similar benefit is also gained by time-segmenting the data used for the deep learning classifier, reducing the complexity of the learned classifiers and increasing the number of available data points for training, and therefore increasing the overall accuracy of the classifiers. In this study, the system will be modular in both sensors and time.

The objective of this study is to utilise deep-learning-driven, time-segmented classification algorithms to develop a system to determine user intent through six sensors across two sensing environments, and to quantify its performance. The work also aims to show the potential of an intent sensing system that is agnostic to the kind of user (abled or disabled). The study will determine the accuracy of the intent prediction for both patients and controls at the early stages of the activity.

## 2. Methods

### 2.1. Data collection

This study uses a novel Parkinson’s disease-based data set (15). Data in this study came from a set of 34 volunteers, 15 of whom had Parkinson’s disease and 19 of whom did not. Demographic information on both the control and patient groups is shown in Table 1, and disease progression information for the patient group is shown in Table 2, including the original 1987 Unified Parkinson’s Disease Rating Scale (UPDRS) (16) and the Hohen and Yahr Stage (17).

**Table 1:** Number of participants, age (mean and standard deviation) and sex for the patient and control groups.

	Patients	Control	Total
Number of participants	15	19	34
Age	67 ± 9	64 ± 10	65 ± 9
Sex	10 Male, 5 Female	11 Male, 8 Female	21 Male, 13 Female

**Table 2:** Disease progression information for the patient group, including duration in years since diagnosis, Unified Parkinson’s Disease Rating Scale (UPDRS) and Hohen and Yahr stage.

Disease duration (years)	5 ± 3
UPDRS	44 ± 19
Hohen and Yahr stage	2 ± 0.5

All metrics include mean and standard deviation.

All volunteers signed an informed consent form and ethical approval for the study was obtained from the NRES Committee South West (REC reference 13/SW/0287). The data collection was performed by research nurses, who supervised the participants throughout the process.

Initially, the participants stood in a calibration pose, with their arms by their sides, with this data recorded for standardization. The participants then performed three standard activities of daily living (ADLs) based on those utilised in the Motor Activity Log, as tested in previous studies (18, 19) – unlocking and opening a door, buttoning and unbuttoning a cardigan, and making toast. Each activity was repeated three times, without a break.

**Figure 1:**
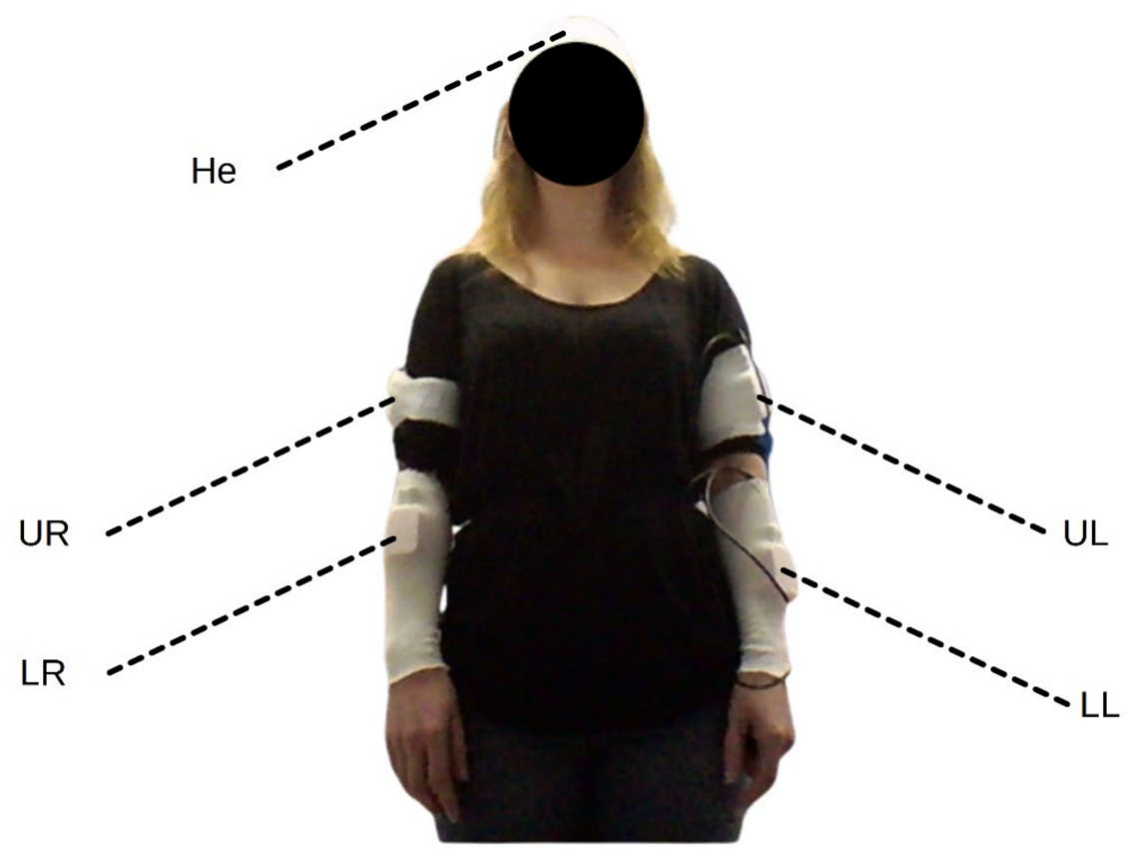
An anonymised participant wearing all five IMU sensors – on the upper left (UL) and right (UR) arm, the lower left (LL) and right (LR) arm, and the head (He).

The participants each wore five Xsens IMU three-axis nine-channel IMUs (MTx, Xsens Technologies B. V., Enschede, Netherlands). These were secured to the participants’ lower and upper arms (both left and right), and to their head (Figure 1). A 44.1 khz microphone on a nearby laptop (Lenovo Thinkpad X1, Dynamic Range 95 dB, Signal-to-Noise Ratio 19 dB) was also used to record audio throughout the activity.

During the activities, the participant was engaged in conversation by the supervising research nurses, but were asked not to talk about the activity they were performing. This engagement was aimed at making the motor behaviour more natural and to better represent activities of daily living in which cognitive loading is increased due to the application of multitasking.

Each IMU provided X, Y, and Z axis data for the magnetometer, gyroscope and accelerometer, along with a 3×3 rotation matrix provided by the XSens software, all at 50 Hz. The microphone positioned in front of the participant provided a 44.1 kHz .wav audio signal.

### 2.2. Processing

The raw 3-axis data from each IMU sensing modality was rotated by the inverse of the mean rotation matrix collected during calibration, to correct for any misalignments in the axes of each sensor. The sensor data was then multiplied by a random rotation for each trial, to prevent the system from being able to use the participant’s starting direction to determine their intent. All data analysis was done using Matlab (MathWorks, Inc., Natick, Massachussetts, United States).

The audio data from the microphone was segmented into groups of 882 samples – 1 group of samples for every single sample from the IMU sensors. This was then processed using a Hamming Window (20) of length 882, followed by Matlab’s AudioFeatureExtractor function to determine the first 18 Mel-Frequency Cepstral Coefficients (MFCCs), which are representations of the power spectrum of the sound (21), for each group. Standard numbers of MFCCs used in similar studies vary between 13 and 25 (22). The number 18 was chosen here in order to match the number of features contributed from each IMU sensor, to ensure the system does not initially weight any one sensor more heavily than the others (weights will be determined and refined during training).

No speech analysis processing was performed, so the system did not attempt to determine the words said during conversation with the supervising nurse, as speech would not be a reliable feature in a real-world scenario where the subject is on their own. Instead, only information about the general nature of the sound, such as power and frequencies, is used – it was anticipated that this would allow detection of events such as the sound of the key turning in the lock, or the buzz of the toaster operating, to more accurately determine the activity.

### 2.3. Deep learning

Time-segmented deep learning was employed to classify the intent as quickly and as accurately as possible. Each trial was divided into time windows of width 500 ms, each with an overlap of 250 ms, enabling a maximum learned pattern length of 250 ms (approximately equal to typical human reaction time (23)). Each time-window was taken as a separate, 108-feature sample for training. Long-Short Term Memory (LSTM) neural networks (24) were trained for each individual sensor, and for all six sensors together. These were trained over 50 epochs (selected experimentally to minimise overfitting and training time), with 15 hidden units, a learn rate of 0.001 and a mini-batch size of 512. Layers consisted of a 108-feature Sequence Input Layer, a single Bidirectional LSTM Layer, a Fully Connected Layer, a Softmax Layer and a Classification Layer. In total, 298.758 min of data were included in the dataset.

Leave-one-out cross-validation was used, such that all the trials for one subject at a time were withheld as a testing set, with the other thirty-three subjects used for training. This was repeated 34 times so that each subject was withheld once, with the results averaged across the set of repeats. To prepare for use in the weighted methods, elaborated on in Section 2.4, this training set was randomly subdivided into a Classifier Learning Set and a Confusion Matrix Learning Set, with half the subjects being included in the former and half in the latter. This was necessary in order to train the LSTM networks for each sensor and then assess their performance both for each sensor and at each time-step, with the results being used to weight the sensor contributions in testing.

A majority voting method was also used which assumed that all sensors and time steps had equal weight – this meant that the full training set could be used to learn the classifier, effectively doubling the size of the training set, at the cost of not being able to have weights specific for each of the sensors.

### 2.4. Modular method

At every time step, each sensor made a prediction using the time-segmented deep-learning method. All the predictions from each sensor at all preceding time steps were then combined. The two weighted methods used Bayes’ Rule, with their contributions effectively being weighted according to the confusion matrices obtained during training. This produced a probability for each of the three possible intents – the intent with the maximum probability was then selected.


(1) $$P\left(E|V\right)=\frac{P\left(V|E\right)\cdot P\left(E\right)}{P\left(V|E\right)\cdot P\left(E\right)+P\left(V|E^{\prime }\right)\cdot P\left(E^{\prime }\right)}$$

P(E) is the prior probability of a particular intent being true, and P(E′) is the prior probability of that intent not being true – in this study, the prior was assumed to be uniform, making P(E) 1/3 and P(E′) 2/3. P(E│V) is the probability of that intent being true given the set of sensor values currently being measured. P(V│E) is the probability of measuring the current sensor values given that the intent being considered is true. Assuming probabilistic independence between the individual sensors, this can be approximated as the product of the probabilities of each individual sensor. P(V│E′) is the probability of measuring the current sensor values given that the intent being considered is not true.

A majority voting method was also tested, effectively giving all sensors and time-steps equal weight. While the loss of the weightings obviously inhibits the ability of the network to incorporate any sensor, no subsets are required within the training set, as there are no confusion matrices to be learned – therefore, a majority voting system will be trained on twice as much data as the weighted methods.

In order to determine how much of the change in accuracy (when using a majority method) comes simply from the access to the larger training set, a majority voting method where half of the training set is discarded is also tested, in order to make it comparable to the weighted methods.

### 2.5. Non-modular method

To provide a comparison, a non-modular method was also used, where the features from all six sensors were included in one single LSTM network. This was also time-segmented.

### 2.6. Comparison

To determine how well the system compares to the theoretically possible accuracies, the naïve model from Russell and Bergmann (14) was used with the confusion matrices obtained during training in order to predict an upper bound for the accuracy of the resultant classifier during testing. The measured accuracy was compared to this upper bound to determine how close performance is to the theoretical maximum.

In this case, the equation for the naïve model is simply:


(2) $$P\left(B\right)=P\left(S_{1}\cup S_{2}\cup \ldots \cup S_{6}\right)=1-P\left(S_{1}\cap S_{2}\cap \ldots \cap S_{6}\right)$$

### 2.7. Monotonic test

As an approximately monotonic increase in accuracy over time is required for an effective intent-sensing system, a Spearman’s Rank test was again applied, quantifying to what extent this requirement was fulfilled by each method.

Where $R\left(P_{i}\right)$ and $R\left(T_{i}\right)$ are the ranks of each (i-th) sample in accuracy and time, respectively, and n is the number of samples, this was calculated using:


(3) $$r_{s}=1-\frac{6\sum {\left(R\left(P_{i}\right)-R\left(T_{i}\right)\right)}^{2}}{n\left(n^{2}-1\right)}$$

This value is always between −1 and 1, where 1 describes a completely monotonically increasing pattern and − 1 describes a perfectly monotonically decreasing pattern. A value of 0 would indicate no monotonic relationship was present (25).

## 3. Results

**Figure 2:**
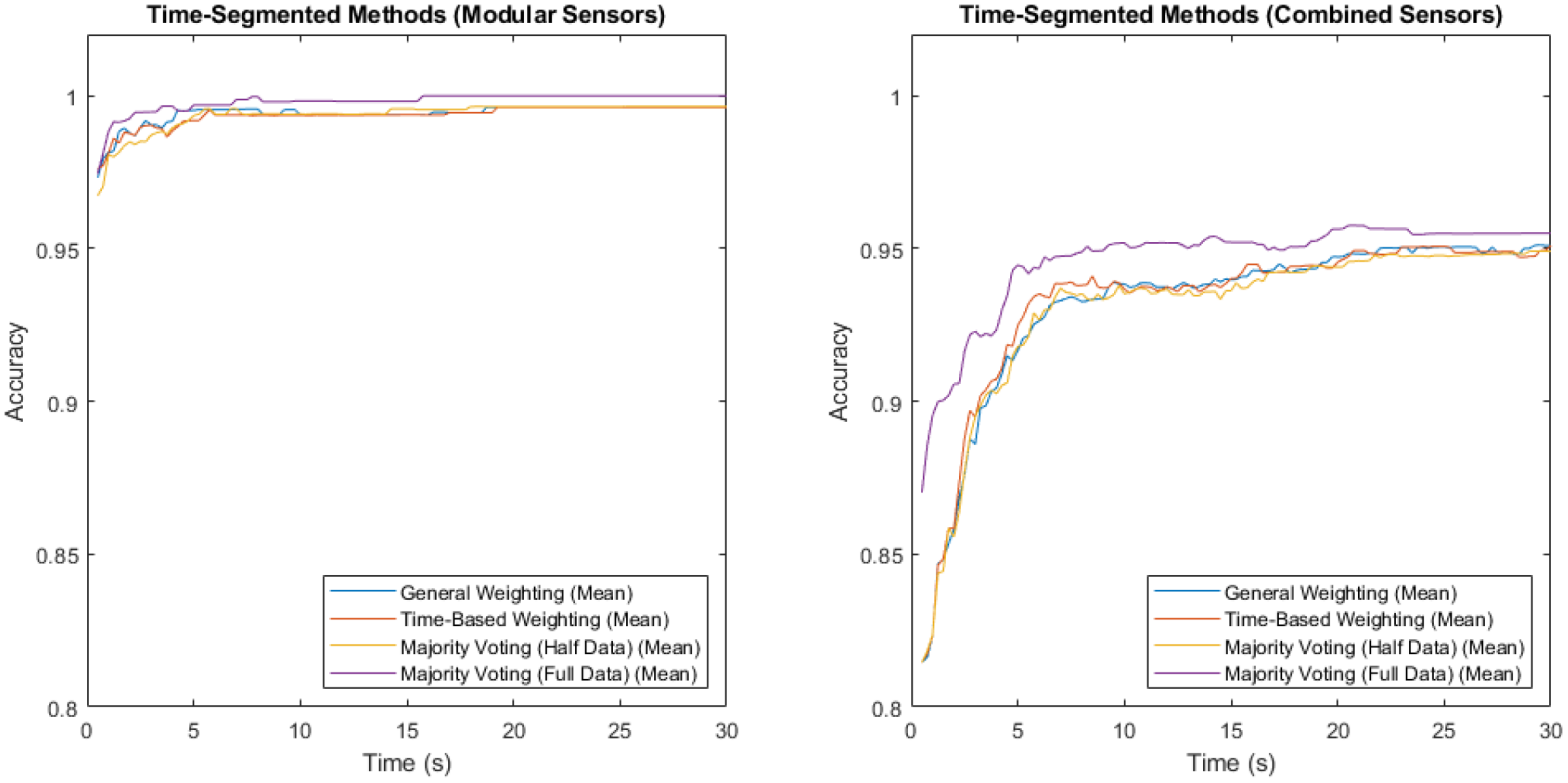
Accuracy vs. time for the modular sensor method and the combined sensor method, using the four different time-segmentation techniques applied across multiple sensing modalities.

Measuring sensor network accuracy using the four time segmentation methods for a system with modular sensors, and for a system with combined sensors, produced Figure 2. Both sets contain data from both the patient and control groups.

The method with the highest accuracy for both of these, at all time steps, was majority voting, where the classifiers were trained on the full data set, but no weightings were used, in both sensors and time. This reached a maximum of 0.999918 for the modular method, and 0.957516 for the combined method.

**Figure 3:**
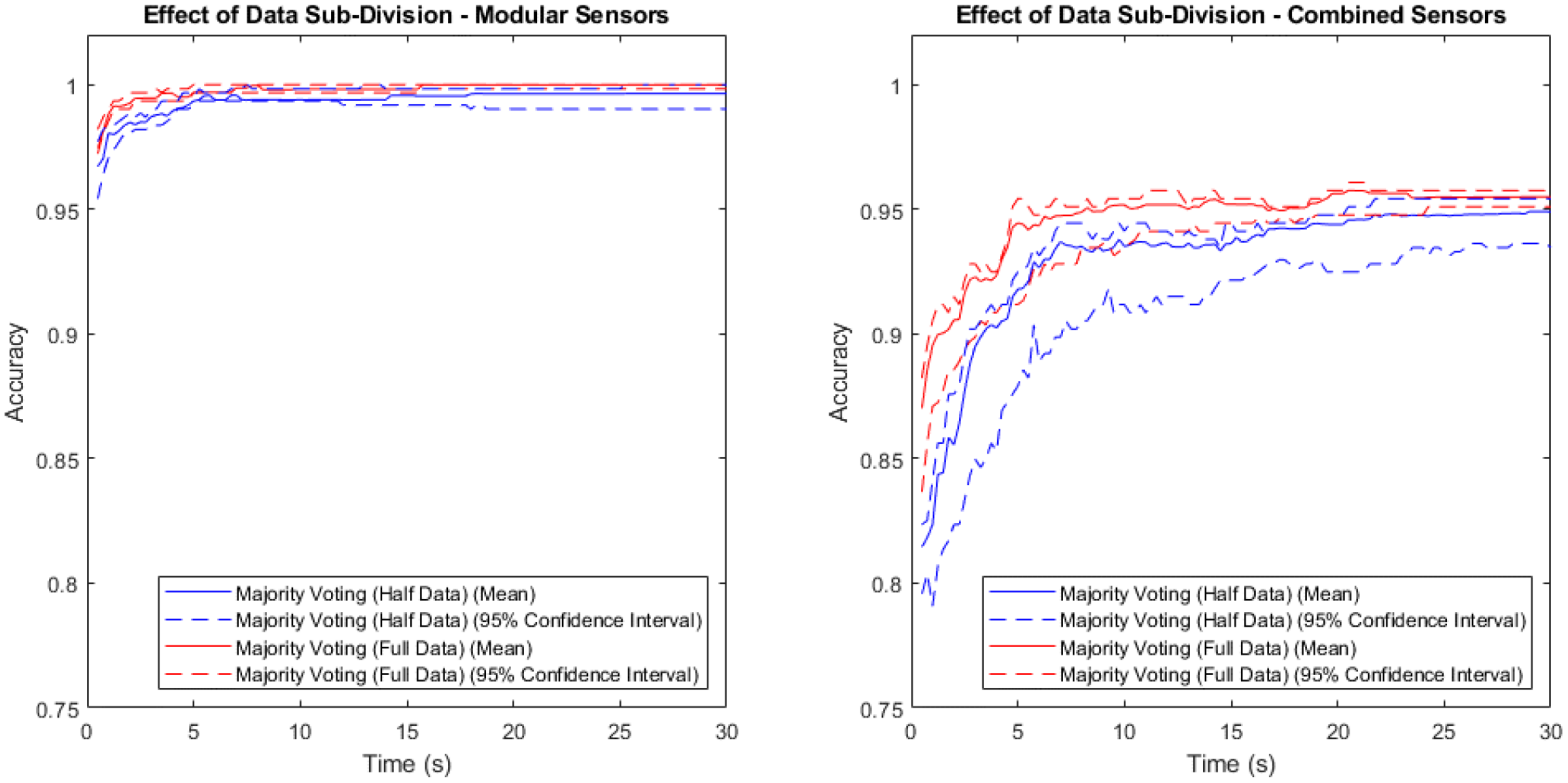
Accuracy vs. time for the modular sensor method and the combined sensor method, combined over time using the majority voting methods with the full and half data sets.

To investigate the extent of the benefit that majority voting gains by training on twice as much data, a comparison of the majority voting method trained on the full data set vs. trained on half the data set is shown in Figure 3. Both sets contain patient, as well as non-patient data.

Both methods showed a higher mean performance with the full data set than with the half data, with a larger difference in the combined method than the modular method.

**Figure 4:**
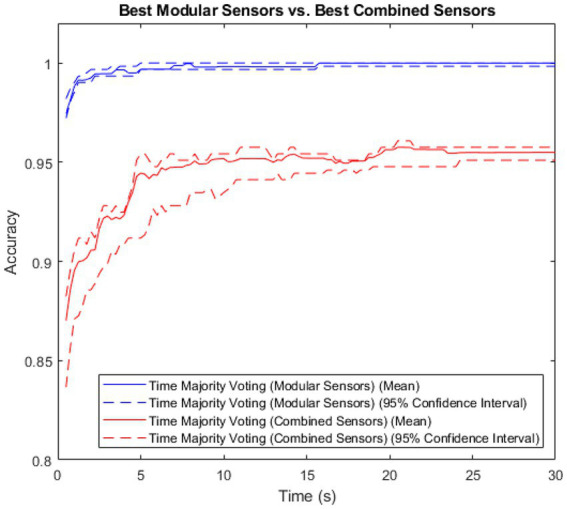
Accuracy vs. time for the modular and non-modular methods, with error shown.

As the majority voting method resulted in the highest accuracy for both methods, it was then used to compare the modular method (sensors) to the non-modular method (sensors), with results shown in Figure 4.

The modular method showed both higher accuracy and lower variance than the combined method. The Spearman’s Rank Coefficient of the modular method was 0.89, and for the non-modular, combined method, 0.62.

**Figure 5:**
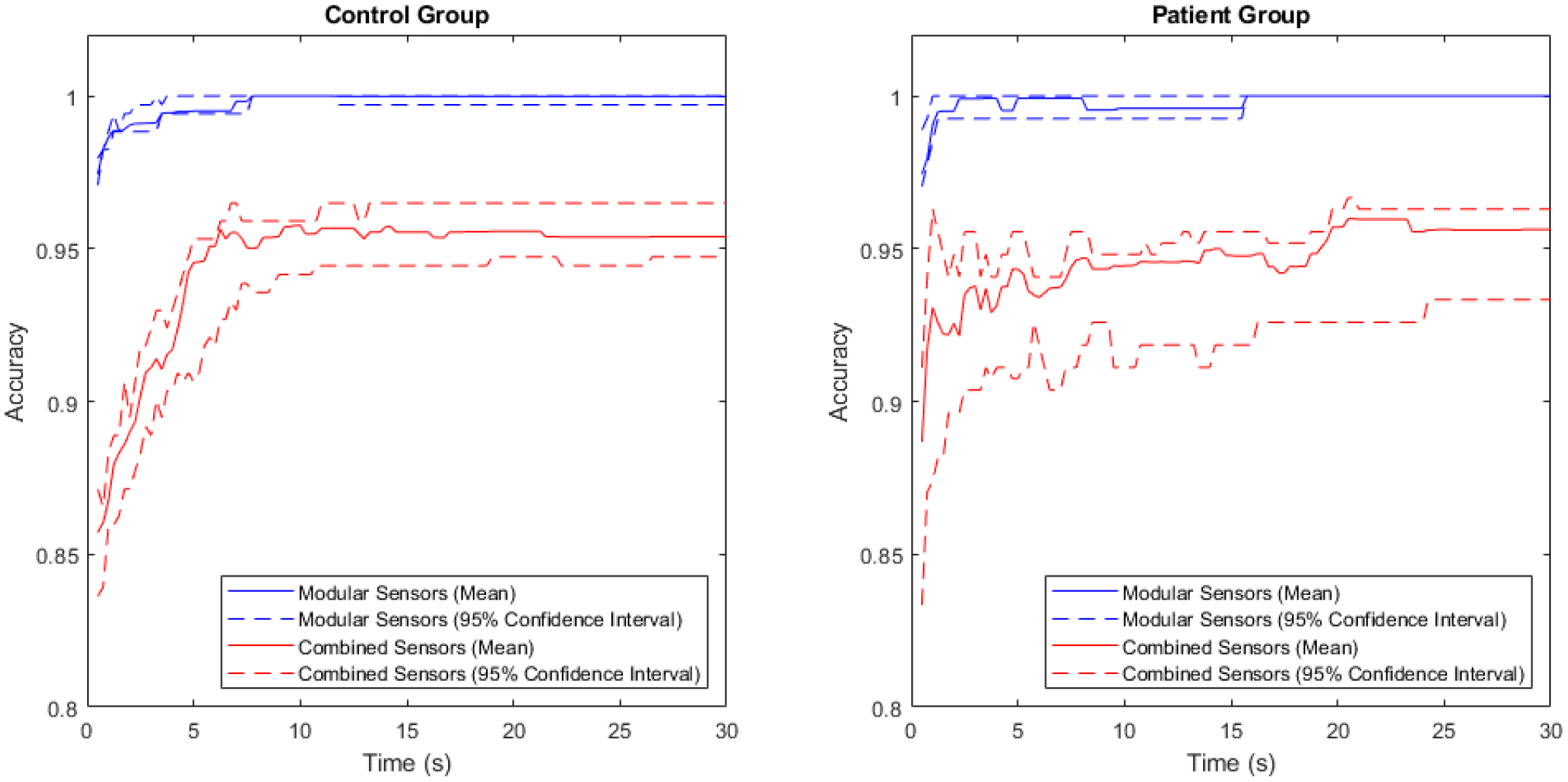
A comparison of accuracy between the modular and combined methods for the patient and control groups, with the central 95% confidence interval plotted for both.

Comparing the accuracy of the modular and non-modular methods across the patient and control groups produced the results shown in Figure 5.

The accuracy of the modular method was consistently high in both groups, rapidly approaching 1. The combined method had a larger variance, and lower maximum accuracy in both groups.

**Figure 6:**
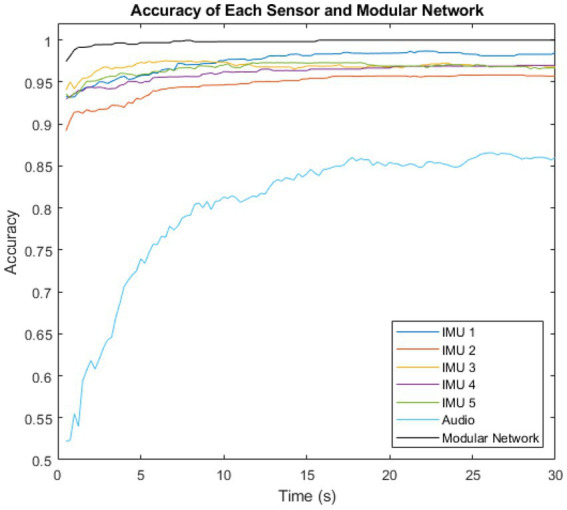
Variation of intent classification accuracy with time after activity inception, shown for the six individual sensors, and for the complete modular intent-sensing network.

The performance of the majority voting (time), modular method (sensors) network was then compared to the performance of each individual sensor. This is shown in Figure 6.

The highest accuracy sensor was IMU 3 in the first 9.5 s, overtaken by IMU 1 for the remaining time. The lowest accuracy sensor was the audio at all time steps.

The theoretical maximum accuracy predicted by the Naïve Model was initially 0.999996 at 0.5 s, which is almost 1. The performance of the modular network method began close to this, at ~0.974. and reached a maximum of 0.999918 within the first 16 s.

## 4. Discussion

### 4.1. Analysis of results

Using the majority voting time-segmented method, trained on the full data set, the modular system was able to classify user intent to an accuracy of 97.4% within only 0.5 s of the inception of the idea to act. This is exceptionally high accuracy, out-performing all previous intent-sensing studies and strongly supporting a modular-sensor, time-segmented deep learning approach for intent classification. While intent is a different goal to activity recognition, these accuracy levels are comparable to those from similar studies, but are achieved in a much faster time (26, 27). This accuracy increased approximately monotonically, with a Spearman’s Rank Coefficient of 0.89, and 16 s after activity inception reached an accuracy of 99.9918%, meaning the system was able to correctly classify almost every trial for every subject by this point.

By comparison, the non-modular, combined method achieved a mean accuracy of only 87.0% in the first 0.5 s, increasing to a maximum of 95.8% after 20.5 s – far lower than the mean accuracy of the modular method. In addition, the Spearman’s Rank Coefficient for this was a lower value of 0.62, suggesting that not only was the accuracy of the non-modular method lower, but it also did not nearly as effectively satisfy the requirement of accuracy increasing monotonically over time.

Figure 6 showed the individual performance of the sensors compared to the overall performance of the modular network. The IMU sensors each showed higher accuracy than the audio. However, the inclusion of the audio modality had the major benefit of it being totally probabilistically independent from the IMU measurements. While the IMUs were located at different sites, they were all constrained by the probability of the sensing environment. A lower bound for this is $P\left(A1\right)=0.987$, the highest accuracy recorded by any of the individual IMU sensors. The highest recorded accuracy for the audio sensor, and therefore the lower bound for the environment probability of audio was $P\left(A2\right)=0.866$. However, as they are both entirely different sensing environments, the overall network is constrained by neither of these limits, and thus outperforms all the individual sensors, and is able to approach 1.

The majority voting method trained on the full data set was once again shown to be the best-performing of the time-segmentation methods trialled. Figures 2, 3 show that this contrast is due to the difference in size of training set, as artificially withholding half of the training set for the majority voting method, in order to make it comparable to the weighted methods, results in very similar recorded accuracies.

The exceptionally high performance of the modular method was observed in both the patient group and the control group, though a 100.00% (to two decimal places) classification accuracy was achieved 8 s later in the patient group than in the control group. This aligned with expectations, as large variations have been observed in the physical activity of patients with Parkinson’s disease (28), suggesting that this would make classifying the patient group harder than classifying the control group.

Even with this difference, the accuracy of classification in the patient group was still very high, strongly supporting intent sensing as a viable method for interpreting user activity. This opens up possibilities for a number of possible clinical applications to support those with Parkinson’s disease, such as assistive exoskeleton technology, which could predict users’ intentions and provide motor support in achieving their task goals that they might not otherwise be able to complete themselves. Alternatively, intent sensing systems could predict activities which might be considered high risk, and rapidly alert carers of the increased possibility of danger to the patient. Intent could also be used as an input for human-computer interfaces, providing more intuitive control to patients over devices which could allow them to communicate and maintain their quality of life as the disease progresses.

The high accuracy in both groups shows the modular intent sensing method as a case example for inclusive design, with ability to apply such a system for both disabled and non-disabled users. Responding to user diversity with appropriate performance across the full range of potential users will bring benefits, such as scalability of technology. An inclusive design approach also provides additional advantages related to desirability and user satisfaction even if their own physical and/or cognitive ability is changing (29).

### 4.2. Limitations of the study

Caution should be taken in the interpretation of these results, as only 3 ADL classes were considered (unlocking and opening a door, buttoning and unbuttoning a cardigan, and making toast). The class prediction is likely to change as more activities are considered – a larger number of classes will lead to a reduced classification accuracy (30).

Additionally, the size of the data set is limited. A future study could be performed with hundreds of participants, increasing the training and testing accuracies and potentially reducing the advantage gained by the majority voting system by using the full data set.

Furthermore, while the wearable IMU system should be applicable in many real-world scenarios, there may be implementation issues with the microphone, the accuracy of which may vary dramatically when used outdoors, or in noisy environments. However, previous studies have shown the proposed sensor fusion algorithm to be robust to sensor dropout (9, 11), meaning that if large amounts of noise are identified, it should be possible to dynamically remove the microphone input from the system. This may also be of benefit if there is any issue with the IMU sensors, such as interfering vibrations from heavy machinery, or a technical fault.

It should also be noted that the activities themselves, while performed without constraints, were using the exact same objects for all volunteers. It has been shown that small changes in objects could lead to different motor patterns (31). Further work is needed to determine the accuracy of this approach in truly free living conditions. Nonetheless, the high accuracy found in this study is promising.

## 5. Conclusion

This study introduces a novel holistic multi-modal intent sensing system. A continuously-updating system was able to predict a user’s intent almost immediately after activity inception, and to continue refining that prediction as time passed. This was done using a modular network of sensors, including two entirely unrelated sensing environments, that might realistically be available to a patient. The system was shown to be highly effective in both the patient and control group, demonstrating it as an effective example of inclusive design.

The results shown in the study highlight intent-sensing as an achievable, highly accurate method of classifying what a user is trying to do, with potential applications in the assessment and support of those with Parkinson’s disease, and throughout many other fields of inclusive design within science and technology.

## Data availability statement

The raw data supporting the conclusions of this article will be made available by the authors, without undue reservation.

## Ethics statement

The studies involving humans were approved by National Research and Ethics Committee South West. The studies were conducted in accordance with the local legislation and institutional requirements. The participants provided their written informed consent to participate in this study.

## Author contributions

JR: Conceptualization, Data curation, Formal analysis, Funding acquisition, Investigation, Methodology, Software, Validation, Visualization, Writing – original draft. JI: Data curation, Investigation, Writing – review & editing. CC: Data curation, Investigation, Writing – review & editing. JB: Conceptualization, Data curation, Funding acquisition, Investigation, Methodology, Project administration, Resources, Supervision, Writing – review & editing.
