# Addendum: A modular, deep learning-based holistic intent sensing system tested with Parkinson's disease patients and controls

**DOI:** 10.3389/fneur.2026.1972445

**Published:** 2026-10-12

**Authors:** Joseph Russell, Jemma Inches, Camille B. Carroll, Jeroen H. M. Bergmann

**Affiliations:** 1 Natural Interaction Lab, Department of Engineering Science, Institute of Biomedical Engineering, University of Oxford, Oxford, United Kingdom; 2 University Hospitals Plymouth NHS Trust, Plymouth, Devon, United Kingdom; 3 Newcastle University Translational and Clinical Research Institute, Campus for Ageing and Vitality, Newcastle-upon-Tyne, United Kingdom; 4 Faculty of Health, University of Plymouth, Plymouth, United Kingdom

**Keywords:** Parkinson's disease, wearable sensors, intent sensing, deep learning, assistive medical devices

An author-narrated audio version of this article is now available on YouTube for readers who prefer to listen rather than read.

You can access it here: https://www.youtube.com/watch?v=08czrhjmnV0&list=PL_Jdsub2aO4UDpUqOy1w_xANulH5Pezzr.

